# An 8-year record of phytoplankton productivity and nutrient distributions from surface waters of Saanich Inlet

**DOI:** 10.1038/s41597-022-01434-y

**Published:** 2022-07-04

**Authors:** Sile M. Kafrissen, Karina E. Giesbrecht, Brandon J. McNabb, Jennifer E. Long, Curtis Martin, Shea N. Wyatt, Marcos G. Lagunas, Diana E. Varela

**Affiliations:** 1 School of Earth and Ocean Sciences, University of Victoria, Victoria, BC Canada; 2 Department of Biology, University of Victoria, Victoria, BC Canada

**Keywords:** Microbial biooceanography, Ecosystem ecology, Marine biology, Marine chemistry, Element cycles

## Abstract

Phytoplankton are the base of nearly all marine food webs and mediate the interactions of biotic and abiotic components in marine systems. Understanding the spatial and temporal changes in phytoplankton growth requires comprehensive biological, physical, and chemical information. Long-term datasets are an invaluable tool to study these changes, but they are rare and often include only a small set of measurements. Here, we present biological, physical and chemical oceanographic data measured periodically between March 2010 and November 2017 from the euphotic zone of Saanich Inlet, a temperate fjord on the west coast of British Columbia, Canada. The dataset includes measurements of dissolved macronutrients, total and size-fractionated chlorophyll-a, particulate carbon, nitrogen and biogenic silica, and carbon and nitrate uptake rates. This collection describes phytoplankton dynamics and the distribution of biologically-available macronutrients over time in the upper water column of Saanich Inlet. We establish a baseline for future investigations in Saanich Inlet and provide a data collection protocol that can be applied to similar productive coastal regions.

## Abstract (summary)

**Table:** 

Measurement(s)	chlorophyll a • biogenic silica • carbon uptake • nitrogen uptake • particulate carbon • particulate nitrogen • inorganic macronutrient dissolved in ocean water • concentration of silicic acid in ocean water
Technology Type(s)	fluorometer • ultraviolet-visible spectrophotometry • elemental analysis and isotope ratio mass spectrometry • nutrient autoanalyzer
Sample Characteristic - Organism	Bacillariophyta • Dinophyceae • Chlorophyceae
Sample Characteristic - Environment	estuary • coastal inlet
Sample Characteristic - Location	Vancouver Island • North East Pacific Ocean coastal waters of Southern British Columbia, Washington and Oregon • Province of British Columbia

## Background & Summary

**Fig. 1:**
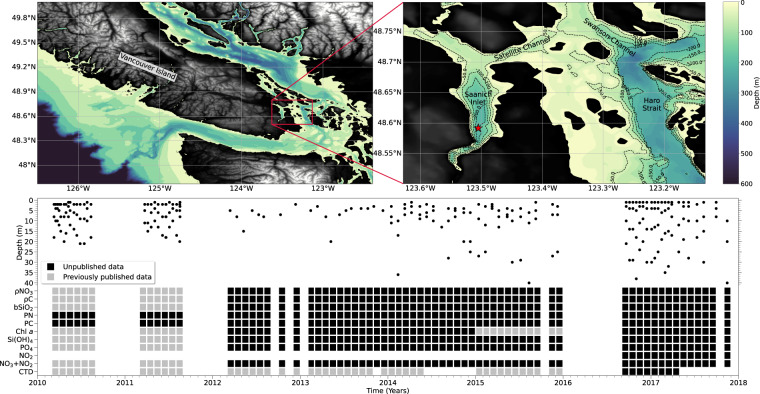
Sampling location, depth of sample collection, and timeline of measurements from March 11, 2010 to November 15, 2017. The upper left panel shows a bathymetric projection of the southern section of Vancouver Island, British Columbia, Canada’s west coast, and surrounding regions. The upper right panel presents a closer look at Saanich Inlet and the sampling location (red star; 48.59°N, 123.50°W). The lower panel shows the sampling depths and timeline for measurements made in the euphotic zone (surface to 40 m); nitrate uptake rate (ρNO_3_), carbon uptake rate (ρC), particulate biogenic silica (bSiO_2_), particulate nitrogen (PN), particulate carbon (PC), Chlorophyll-a (Chl-a), silicic acid (Si(OH)_4_), dissolved phosphate (PO_4_^3−^), dissolved nitrite (NO_2_^−^), and dissolved nitrate plus nitrite (NO_3_^−^ + NO_2_^−^). CTD measurements include depth, temperature and conductivity, with the addition of photosynthetically active radiation (PAR), fluorescence and dissolved oxygen when available. Data presented for the first time in this publication are represented by black squares, while previously published data^20,37,38^ included in this data record are shown with grey squares. The year labels are positioned under the tick marks corresponding to January. Table 1 lists all measurements available in the associated data file and figures.

Saanich Inlet is a 24-km long temperate fjord located on south-eastern Vancouver Island in British Columbia, Canada (Fig. 1). The inlet is characterized by strong vertical biogeochemical gradients^1^ and high levels of primary productivity^2–4^. At its deepest location, Saanich Inlet reaches a depth of ~215 m, but a shallow (~70 m) glacial sill at the mouth of the inlet restricts mixing and exchange of deep waters^5^. Unlike most estuaries, the dominant freshwater input to Saanich Inlet occurs at the mouth, from the Fraser and Cowichan Rivers, whereas discharge from Goldstream River, located at the head of the inlet, is very low^5^. These freshwater inputs create a reverse estuarine flow regime where freshwater primarily moves into the inlet across the mouth at the surface, and deeper saltier water flows out of the mouth at sill depth^2^. Most vertical mixing in Saanich Inlet is due to tidally-driven pressure gradients that form during the 14-day spring-neap tidal cycle. This tidal forcing allows for brief periods of normal estuarine flow and the injection of sub-surface, nutrient-rich waters into the euphotic zone^2,6,7^.

Due to high pelagic primary productivity, efficient downward matter flux, and infrequent renewal of deep waters, the deep basin of Saanich Inlet regularly becomes anoxic^1,2,4,8^. Deep waters are renewed when cold, upwelled waters from the west coast of Vancouver Island (Fig. 1) are able to flow through Haro Strait and over the sill; a process that typically occurs during the late summer and early fall, if at all^1,8,9^. The sporadic renewal of deep waters in Saanich Inlet contributes to deep-water reduction-oxidation (redox) conditions that make Saanich Inlet an ideal location for investigating the biological and chemical effects of expanding hypoxic marine environments and oxygen minimum zones (OMZs)^10,11^. Saanich Inlet is surrounded by farm land, industries and urban development from where runoff and discharge can lead to localized high nutrient inputs. It is therefore important to establish baselines for nutrient budgets as well as primary producer biomass and productivity rates to understand future impacts of anthropogenic activities and climate change on this and other similar coastal system.

One of the dominant groups of pelagic primary producers in Saanich Inlet are diatoms^12^, which tend to proliferate in productive, temperate ecosystems, such as major upwelling regions and coastal fjords^13–16^. As a relatively large and nutritionally high-quality phytoplankton, diatoms are important for supporting marine food webs with high animal biomass in Saanich Inlet and elsewhere. These animals include several economically important species such as Pacific herring (*Clupea pallasii)* and Pacific salmon (*Oncorhynchus sp.)*. Due to their obligate silicon (Si) requirement to build their silica (SiO_2_) frustules, diatoms need to take up dissolved Si in the form of silicic acid (Si(OH)_4_). Measurements of suspended particulate biogenic silica (bSiO_2_) in seawater provide estimates of siliceous phytoplankton biomass (primarily diatoms) and their contribution to total phytoplankton biomass. By comparing changes in diatom biomass to oceanographic conditions and nutrient availability, we can better understand how processes such as eutrophication and climate change may affect marine ecosystems in the inlet.

Fluctuating deep-water hypoxia, high pelagic primary productivity, restrictive bathymetry, and exposure to human activities result in a seasonally variable biogeochemical environment in Saanich Inlet^2,5,17,18^. This fjord provides a useful natural laboratory to investigate the relationships between primary productivity, concentrations and distributions of macronutrients, and abiotic oceanographic factors. The 2010–2017 data presented in this paper can be particularly useful when combined with other publicly available Saanich Inlet datasets such as those generated by the Ocean Networks Canada cabled observatory^19^, and by other groups^20,21^. Previous measurements (2005–2006) conducted in Saanich Inlet are not included here, but are available in an earlier manuscript from our research group^4^. The data from 2016 to 2017 were collected as part of the Saanich Inlet Redox Experiment (SaanDox) lead by Dr. Roberta Hamme at the University of Victoria. All samples and data were obtained and analyzed by members of Dr. Diana Varela’s laboratory at the University of Victoria over almost eight years using consistent methodologies. This long time-series of observations can be extrapolated to understand how primary producers are involved in larger oceanic processes such as the formation of OMZs, eutrophication, carbon export, and fisheries production in other marine systems.

## Methods

### Sample collection and hydrography

Sampling was conducted aboard the University of Victoria's *MSV John Strickland* either weekly, biweekly or monthly between 11 March 2010 and 15 November 2017 in Saanich Inlet at 48.59°N, 123.50°W (Fig. 1). To standardize measurements and due to biological significance, seawater was collected from the euphotic zone. Sampling depths corresponded to approximately 100, 50, 15, and 1% of the photosynthetically active radiation (PAR) at the surface (I_o_). These “light” depths were either determined using a CTD-mounted PAR sensor or a Secchi disk. CTD profiles were performed prior to each seawater cast to measure depth, temperature and conductivity of the water column, and PAR, fluorescence, and dissolved oxygen (when available).

Seawater from each light depth was collected using Niskin or GO-FLO bottles on either a rosette sampler or an oceanographic wire. When possible, individual samples were collected directly from the Niskin or GO-FLO bottles. When time was not sufficient to allow direct sampling, bulk samples of seawater from each depth were collected into acid-washed polyethylene carboys, kept cold in the dark, and homogenized before sub-sampling for the individual measurements.

### Dissolved nutrients

**Fig. 2:**
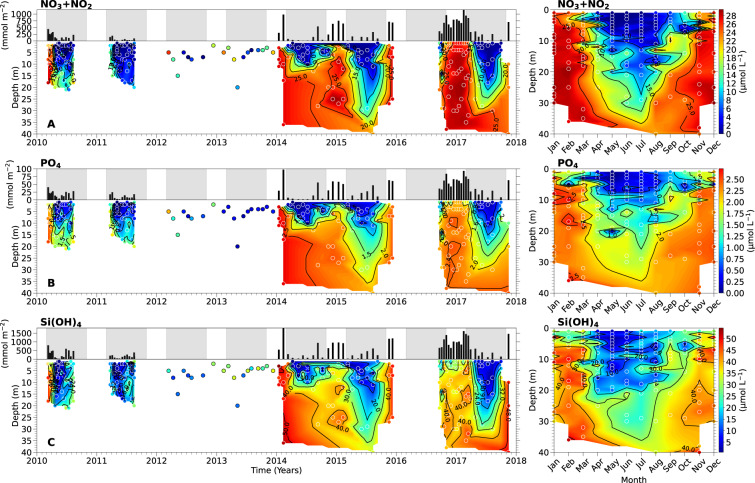
Dissolved macronutrient concentrations in the euphotic zone of Saanich Inlet from March 11, 2010 to November 15, 2017. Left panels show depth-integrated concentrations (black bars on top) and time-series profiles (filled contour/scatter plots on bottom) for (**A**) nitrate plus nitrite (NO_3_^−^ + NO_2_^−^), (**B**) phosphate (PO_4_^3−^) and (**C**) silicic acid (Si(OH)_4_). In the time-series profiles, 2012–2013 data are not interpolated due to single-depth sampling. Grey shaded regions in top panels indicate the phytoplankton growing seasons considered for this study (March 1^st^ – October 30^th^). Right panels show monthly-averaged depth profiles for the entire 8-year period, illustrating euphotic zone seasonality for each nutrient. The color scale bars on the far right apply to both the time-series vertical profiles and the 8-year seasonal plots. Sampling depths are indicated by round symbols. The year labels are positioned under the tick marks corresponding to January.

For the measurements of nitrite (NO_2_^−^), nitrate and nitrite (NO_3_^−^ + NO_2_^−^), phosphate (PO_4_^3−^) and Si(OH)_4_, seawater samples from each light depth were syringe-filtered through a combusted 0.7 µm (nominal porosity) glass fibre filter into acid-washed 30-mL polypropylene bottles and immediately frozen. All nutrient samples were stored at −20 °C until analysis. Concentrations of NO_2_^−^, NO_3_^−^ + NO_2_^−^, PO_4_^3−^, and Si(OH)_4_ were determined using an Astoria Nutrient Autoanalyzer (Astoria-Pacific, OR, USA) following the methodology of Barwell-Clarke and Whitney^22^. During 2014 and 2015, samples for the measurement of Si(OH)_4_ were collected separately from those for the other nutrients, filtered with a 0.6 µm polycarbonate membrane filter and stored at 4 °C. During this period, Si(OH)_4_ concentrations were determined manually using the molybdate blue colorimetric methodology^23^. Replicate (2 or 3) nutrient samples were taken at each depth; average data are presented in the published dataset and the figures (Fig. 2).

### Suspended particulate matter

#### Total chlorophyll-a

**Fig. 3:**
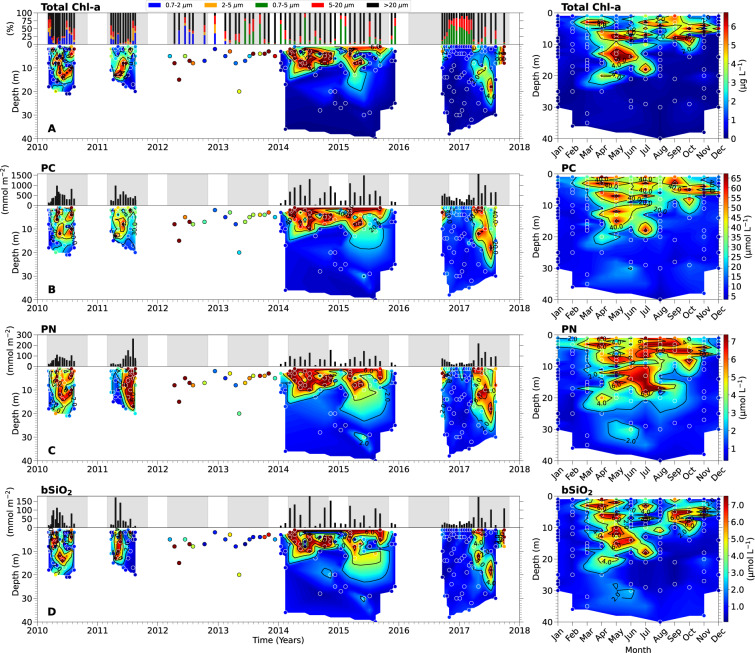
Biological particulate concentrations in the euphotic zone of Saanich Inlet from March 11, 2010 – November 15, 2017. Left panels show time-series profiles (filled contour/scatter plots) of (**A**) total chlorophyll-a (Total Chl-a), (**B**) particulate carbon (PC), (**C**) particulate nitrogen (PN), and (**D**) particulate biogenic silica (bSiO_2_). The 2012–2013 data are not interpolated due to single-depth sampling. In **A**, the bar plot in the top panel shows percent contribution of different size fractions to total Chl-a. In (**B**–**D**), black bars in top panels show depth-integrated concentrations. Grey shaded regions in bar plots indicate phytoplankton growing seasons considered for this study (March 1^st^ – October 30^th^). Right panels show monthly-averaged depth profiles for the entire 8-year period, illustrating euphotic zone seasonality for each particulate. The color scale bars on the far right apply to both the time-series vertical profiles and the 8-year seasonal plots. Sampling depths are indicated by round symbols. The year labels are positioned under the tick marks corresponding to January.

Chlorophyll-a (Chl-a) was used as a proxy for phytoplankton biomass (Fig. 3A). For total Chl-a analysis, seawater samples (0.25–1 L) were gently vacuum filtered onto 0.7 µm (nominal porosity) glass fiber filters, which were then stored at −20 °C until analysis. Chl-a concentrations were determined using the acetone extraction and acidification method^24,25^. Acidification of samples decreased the likelihood of overestimation of Chl-a concentrations due to the presence of chlorophyll degradation products^26^. Filters were submerged in 10 mL of 90% acetone, sonicated for 10 minutes in an ice bath, and left to extract at −20 °C for 22 h. Following the extraction period, samples were allowed to equilibrate to room temperature (~2 h). Fluorescence of the acetone solution containing the extracted Chl-a was measured before and after acidification with 1.2 N hydrochloric acid using a Turner 10-AU fluorometer. The final concentrations of total Chl-a were calculated from measurements made before (Fo) and after (Fa) acidification using Eq. (1)^25^. The coefficient (τ) of Eq. (1), adapted from Strickland and Parsons^25^, was derived from a calibration of the Turner 10-AU fluorometer with known pure chlorophyll standards (Table 2).

1 $${\rm{C}}{\rm{h}}{\rm{l}} \mbox{-} {\rm{a}}(\mu g\,{L}^{-1})=\frac{\tau }{\tau -1}\ast ({\rm{F}}{\rm{o}}-{\rm{F}}{\rm{a}})\ast 0.814\ast \left(\frac{{\rm{V}}{\rm{o}}{\rm{l}}.{\rm{A}}{\rm{c}}{\rm{e}}{\rm{t}}{\rm{o}}{\rm{n}}{\rm{e}}\,{\rm{e}}{\rm{x}}{\rm{t}}{\rm{r}}{\rm{a}}{\rm{c}}{\rm{t}}{\rm{e}}{\rm{d}}}{{\rm{V}}{\rm{o}}{\rm{l}}.{\rm{S}}{\rm{e}}{\rm{a}}{\rm{w}}{\rm{a}}{\rm{t}}{\rm{e}}{\rm{r}}\,{\rm{f}}{\rm{i}}{\rm{l}}{\rm{t}}{\rm{e}}{\rm{r}}{\rm{e}}{\rm{d}}}\right)$$

#### Size fractionated chlorophyll-a

To determine the percent contributions of “pico” (0.7–2 µm), “small nano” (2–5 µm), “large nano” (5–20 µm) and “micro” (>20 µm) phytoplankton to total Chl-a, seawater samples (0.25–1 L) separate from those used for total Chl-a) were consecutively filtered through 20, 5 and 2 µm polycarbonate membrane filters and 0.7 µm (nominal porosity) glass fiber filters. Between 2013 and 2017, the “pico” and “small nano” size classes were collected as one fraction (0.7–5 µm). Analysis of Chl-*a* concentrations for each size fraction followed the same procedure outlined for total Chl-a.

### Particulate carbon and nitrogen

Particulate C and N measurements were obtained from seawater samples incubated for carbon (ρC) and nitrate uptake (ρNO_3_) rates (see section on “Uptake rates of carbon and nitrate” for methodology) (Fig. 3B,C). PC and PN measurements presented in this dataset were taken at the end of ρC and ρNO_3_ incubations; however, original (‘ambient’) values can be back calculated by subtracting the amount of C and N taken up during the incubation period from the final PC and PN values. The differences between after-incubation PC and PN data and back-calculated ambient values were not significantly different than the measurement error.

### Particulate biogenic silica

Particulate biogenic silica was used as a proxy for siliceous phytoplankton biomass (Fig. 3D). Seawater samples (0.5–1 L) from each depth were gently vacuum filtered through 0.6 µm polycarbonate membrane filters. Filters were folded and placed in polypropylene centrifuge tubes, dried for 48 h at 60 °C, and then stored in a desiccator at room temperature until analysis. Filters were digested with 4 mL of 0.2 M NaOH for 30–45 min in a water bath at 95 °C^27^. After digestion, samples were neutralized with 0.1 N HCl and cooled rapidly in an ice bath. Samples were centrifuged to separate out the undissolved lithogenic silica, and colorimetric analysis was performed on the supernatant. The transmittance of the samples, standards, and reverse-order reagent blanks were read at 820 nm using a Beckman DU 530 ultraviolet-visible (UV/Vis) spectrophotometer^27,28^.

### Uptake rates of carbon and nitrate

Seawater samples (~0.5–1 L) were gently collected into clear polycarbonate bottles. One additional sample was collected from the 100% light depth, into a dark polycarbonate bottle, which did not allow light penetration. After the addition of the isotopic tracers (see below), bottles were placed into an acrylic incubator with constant seawater flow to maintain surface seawater temperature. Three acrylic tubes wrapped in colored and neutral density photo-film (to obtain 50, 15, and 1% of surface PAR) were used to incubate sample bottles under the same *in-situ* light conditions from which samples were collected. Samples from the 100% light level were placed inside the same acrylic incubator, but outside of the film-covered tubes. A LI-COR® LI-190 Quantum sensor was installed next to the incubator and continuously recorded incoming PAR for the entire incubation period. During sampling in 2010 and 2011, all experiments were performed using a shipboard incubator. For sampling from 2012 onwards, all experiments were done using an incubator on land (University of Victoria Aquatic Facility), which was connected to a seawater system maintained at local surface seawater temperature (approximately 9–12 °C depending on the time of year).

**Fig. 4:**
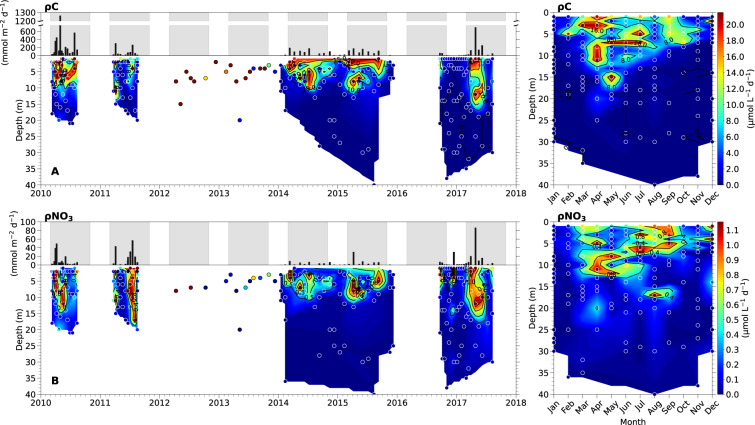
Carbon and nitrate uptake rates in the euphotic zone of Saanich Inlet from March 11, 2010 – November 15, 2017. Left panels show depth-integrated rates (black-bars on top) and time-series profiles (filled contour/scatter plots below) of (**A**) carbon (*ρ*C) and (**B**) nitrate (*ρ*NO_3_) uptake rates. In the time-series profiles, 2012–2013 data are not interpolated due to single-depth sampling. Grey shaded regions in depth-integrated plots indicate phytoplankton growing seasons for this study (March 1^st^ – October 30^th^). Right panels show monthly-averaged depth profiles for the entire 8-year period, illustrating euphotic zone seasonality for carbon and nitrate uptake. The color scale bars on the far right apply to both the total time-series vertical profiles and the 8-year seasonal plots. Sampling depths are indicated by round symbols. The year labels are positioned under the tick marks corresponding to January.

Rates of C (ρC) and NO_3_ (ρNO_3_) uptake were determined using a stable isotope tracer-technique^29,30^ (Fig. 4). A single seawater sample from each light depth received a dual spike, with NaH^13^CO_3_ (99% ^13^C purity, Cambridge Isotope Laboratories) for the determination of ρC and Na^15^NO_3_ (98 + % ^15^N purity, Cambridge Isotopes Laboratories) for the determination of ρNO_3_. Isotope additions were made at approximately 10% of ambient dissolved inorganic carbon (DIC) and NO_3_^−^ concentrations.

Spiked seawater samples were incubated for 24 h, except from 2010 to 2013 when the incubation period was 4 to 6 hr. After incubation, the entire sample was gently vacuum filtered onto a combusted 0.7 µm (nominal porosity) glass fibre filter. Filters were dried for 48 h at 60 °C and kept in a desiccator at room temperature until analysis. Filters were packed into pellets and sent to the Stable Isotope Facility at the University of California (UC) Davis for analysis of ^13^C and ^15^N enrichment, and total C and N content by continuous flow isotope ratio mass spectrometry and elemental analysis, respectively. For these measurements, UC Davis uses either an Elementar Vario EL Cube or Micro Cube elemental analyzer (Elementar Analysensysteme GmbH, Hanau, Germany) interfaced to either a PDZ Europa 20–20 isotope ratio mass spectrometer (Sercon Ltd., Cheshire, UK) or an Isoprime VisION IRMS (Elementar UK Ltd, Cheadle, UK).

Carbon and NO_3_^−^ uptake rates were calculated using Eq. 3 of Hama *et al*.^29^, and Eq. 3 and 6 of Dugdale and Wilkerson^30^, respectively.

For samples incubated for less than 24 h, the daily C or NO_3_^−^ uptake rates (ρX) were calculated using a PAR extrapolation method shown in Eq. (2):

2 $$\rho X(\mu mol\,{L}^{-1}da{y}^{-1})=\left(\rho X(\mu mol\,{L}^{-1}h{r}^{-1})\div\left(\frac{PAR\,during\,incubation}{Total\,Daily\,PAR}\right)\right)\ast 24$$

Additionally, to account for NO_3_^−^ uptake occurring under no light, ρNO_3_^−^ was measured in dark bottles and this rate was added to the ρNO_3_^−^ of each sample incubated for less than 24 h. The ρNO_3_^−^ DARK was calculated following Eq. (3):

3 $$\rho N{O}_{3}DARK(\mu mol\,{L}^{-1}da{y}^{-1})=\left(\rho N{O}_{3}DARK(\mu mol\,{L}^{-1}h{r}^{-1})\div\left(\frac{Total\,Daily\,PAR-PAR\,during\,incubation}{Total\,Daily\,PAR}\right)\right)\ast 24$$

PAR data used in Eqs. (2) and (3) came from the LI-COR® LI-190 Quantum sensor that was mounted beside the incubator. The seawater DIC value for each sample was calculated using a regression equation relating water density to DIC for Saanich Inlet^31^. Ambient NO_3_^−^ concentrations were measured as described above.

## Data Records

The Saanich Inlet biological dataset is accessible as a tab delimited data file “Saanich_BioOcean_Data.tab” (10.5683/SP2/6BATWK) on Dataverse^32^ and contains the data fields outlined in Table 1. Data is presented for discrete depths of all measurements and as depth integrated values for dissolved nutrient, biomass and nutrient uptake  rates.

**Table 1:** Key to the data fields in the Saanich Inlet Biological Dataset.

Data field	Description	Units	Integrated Units
Date	Cruise and samples collection date	YYYY-MM-DD	
Time of Sampling	Time of the day of seawater collection	Pacific Time	
Time of incubation start	Time of the day of placement of samples for C and N uptake in incubator	Pacific Time	
LAT	Latitude of sample collection	Degrees N	
LON	Longitude of sample collection	Degrees W	
Light Penetration	Percentage of surface PAR measured at sampling depth	%	
Depth	Depth of sample collection	Meters (m)	
Temperature	Water temperature from CTD	Degrees Celsius	
Salinity	Practical salinity from CTD	PSU	
Fluorescence	Total fluorescence from CTD	mg m^−3^	
O_2_	Dissolved oxygen from CTD	mL L^−1^	
NO_2_^−^	Dissolved nitrite (NO_2_)	µmol L^−1^	
NO_3_^−^ + NO_2_^−^	Dissolved nitrate (NO_3_) and nitrite (NO_2_) presented as one value	µmol L^−1^	mmol m^−2^
PO_4_^3−^	Dissolved phosphate	µmol L^−1^	mmol m^−2^
Si(OH)_4_	Dissolved silicic acid	µmol L^−1^	mmol m^−2^
Chl-a (> 20 µm)	Contribution to total chlorophyll-a by the >20 µm size fraction	% contribution	mg m^−2^
Chl-a (5–20 µm)	Contribution to total chlorophyll-a by the 5–20 µm size fraction	% contribution	mg m^−2^
Chl-a (2–5 µm)	Contribution to total chlorophyll-a by the 2–5 µm size fraction	% contribution	mg m^−2^
Chl-a (0.7–2 µm)	Contribution to total chlorophyll-a by the 0.7–2 µm size fraction	% contribution	mg m^−2^
Chl-a (0.7–5 µm)	Contribution to total chlorophyll-a by the 0.7–5 µm size fraction	% contribution	mg m^−2^
Total Chl-a	Total concentration of chlorophyll a (phytoplankton larger than 0.7 µm in diameter)	µg L^−1^	mg m^−2^
bSiO_2_	Concentration of biologically-derived particulate silica (SiO_2_)	µmol L^−1^	mmol m^−2^
PC	Total particulate carbon	µmol L^−1^	mmol m^−2^
PN	Total particulate nitrogen	µmol L^−1^	mmol m^−2^ day^−1^
rho_C	Rate of carbon uptake derived from using ^13^C tracer (ρC)	µg C L^−1^ day^−1^ and µmol C L^−1^ day^−1^	mmol C m^−2^ day^−1^
rho_NO_3_	Rate of nitrate uptake derived from using ^15^N tracer (ρNO_3_)	µmol N L^−1^ day^−1^	mmol N m^−2^ day^−1^

## Technical Validation

### Sample collection and analyses

All seawater samples were collected by qualified individuals aboard the University of Victoria’s *MSV John Strickland.* Personnel were trained either by experienced members of Dr. Varela’s lab or by Dr. Varela herself at the University of Victoria before participating in research cruises, conducting sample collection or analysing samples using well-established protocols. Seawater was always collected into clean, acid-washed bottles to prevent contamination, except for Chl-a samples for which clean, non-acid washed bottles were used. Samples were kept cold and in the dark immediately after collection, and were processed as soon as possible.

### Calibration of CTDs

Two CTDs were used over the course of the study. During 2010–2011 and 2016–2017, a Seabird SBE 19plus CTD equipped with a SBE 43 Oxygen sensor and a WETLabs fluorometer was used. The Seabird SBE 19plus CTD was returned to the manufacturer every two years for the calibration of all sensors. During 2012–2015, a Seabird 43 CTD equipped with a Biospherical PAR sensor was used in collaboration with Dr. Steve Hallam’s laboratory at the University of British Columbia, Vancouver, BC, Canada. Information about the calibration of the Seabird 43 CTD is described by Torres-Beltrán *et al*.^20^.

### Precision of dissolved nutrient data and sample preservation techniques

The limits of detection for NO_3_^−^ + NO_2_^−^, PO_4_^3−^ and Si(OH)_4_ were 0.1 µmol L^−1^, 0.03 µmol L^−1^ and 0.2 µmol L^−1^, respectively. The average coefficients of variation (CV) for these nutrients were 9% for NO_3_^−^ + NO_2_^−^_,_ 6% for PO_4_^3−^_,_ and 8% for Si(OH)_4._

Filtration of samples for Si(OH)_4_ from 2010 to 2013, in 2016 and in 2017 was done with combusted 0.7 µm glass fiber filters and stored at −20 °C (Si(OH)_4Frozen_) and analyzed in the nutrient autoanalyzer alongside the other nutrients following Barwell-Clark and Whitney^22^ protocols. Filtration of Si(OH)_4_ samples in 2014 and 2015 was done with 0.6 µm polycarbonate filters, stored at 4 °C (Si(OH)_4Cold_) and analyzed manually^23^. A direct comparison between these sampling and storage methods showed that [Si(OH)_4_]_cold_ were more precise^33^. This difference was especially pronounced when [Si(OH)_4_] were >15 µmol L^−1^ and resulted in [Si(OH)_4_]_frozen_ being ~16% lower than [Si(OH)_4_]_cold_, on average. However, given that this difference is within the average standard deviation for replicates for [Si(OH)_4_]_frozen_, the data presented here were not corrected.

### Calibration of fluorometer for chlorophyll-a measurements

The same Turner 10-AU fluorometer was used to measure all Chl-a samples over the course of the study. The fluorometer was calibrated three times during the study period using standards of pure Chl-*a* extracted from the algae *Anacystis nidulans* and the working coefficient (τ) was updated after each calibration (Table 2). Calibration of the fluorometer was frequently checked between calibration dates by measuring solid Chl-a standards provided by Turner Designs®.

**Table 2:** Fluorometer (Turner Designs®, Model 10-AU) calibration dates and value of coefficient (τ) used in Eq. (1) for the calculation of chlorophyll-a during this study.

Date Calibrated	Coefficient (τ)
Dec 1, 2008	1.991
April 12, 2012	2.024
Aug 24, 2015	2.038

### Precision of particulate biogenic silica data and sample digestion

At each sampling time triplicate (n = 3) samples of bSiO_2_ were collected at 100% I_o_ and were used to calculate a coefficient of variation (CV). The CV of 12.9% ± 7.9 is representative of the bSiO_2_ values presented in this dataset. Single measurements were conducted at depths below 100%.

The NaOH digestion method used for the determination of bSiO_2_ in this study may result in the digestion of 10–15% of lithogenic SiO_2_ from the samples^34^. Digestion times were chosen as to limit the likelihood of lithogenic SiO_2_ leaching while at the same time, completely digesting bSiO_2_.

### Carbon and nitrogen techniques: isotopic ratios and particulates

Vienna Pee Dee Belemnites and Air were used as the international reference standards for ^13^C and ^15^N measurements, respectively. Details on the specific protocols for instrument calibration at UC Davis can be found here: https://stableisotopefacility.ucdavis.edu/carbon-and-nitrogen-solids.

The material collected on the filters at the end of the incubations was not acidified before measuring the stable isotope ratios and PC/PN concentrations at UC Davis. Therefore, the total PC after the incubation could potentially include inorganic CaCO_3_ material that can overestimate the total organic C content of the sample. Similarly, ^13^C uptake rates could also be overestimated by the activity of coccolithophores. Although we did not conduct direct measurements of calcifying material in the water column or compare rates before and after acidification, it is known that Saanich Inlet is a diatom dominated system and that there is little to no contribution from calcifying phytoplankton^12,35^.

### Incubation methodology

To minimize the loss of particulate matter due to adsorption on bottle walls during the incubation, samples were vigorously rinsed three times with filtered seawater and the rinse water was also filtered through the same filter at the end of the incubation period^36^. Additionally, “blank” and “dark” rates were measured with each set of incubations. Blank samples were enriched with isotopes but were immediately filtered onto combusted 0.7 µm glass fibre filters. Dark samples were enriched with isotopes and incubated in completely black bottles. Data from the blank samples provided the natural isotopic values of ^13^C and ^15^N used in the calculation of uptake rates. Dark bottle ^13^C values were used as a validation tool in order to confirm that there was no measured ^13^C uptake under no-light conditions. Dark bottle ^15^NO_3_ values were used to correct for daily rates of ^15^NO_3_^−^ uptake for <24 h incubations (see above). Incubations were maintained at approximately surface seawater temperature for the length of the incubation periods. The average temperature difference between the surface and the deepest depth sampled was <2 °C.

## Usage Notes

Data are presented in a single tab delimited excel file. Discrete depth concentrations are presented in the excel file followed by integrated total euphotic zone values. Values below the limit of detection are presented as 0 in this dataset but may be interpreted as any number between 0 and the limit of detection defined in this manuscript for each parameter measured.

Partial CTD, nutrient and biomass data from 2010, 2011, and 2015 have been included, as averages, in two previously published studies^37,38^. Here, we include the complete data for each depth for the 2010–2011, and 2015 sampling period. Additionally, some CTD data collected between 2012–2015 in collaboration with Dr. Hallam’s lab at UBC, have been previously published^20^ but are included here for completeness of the dataset. CTD data from 2016 and 2017 presented here were collected as part of the Saandox project and have not been previously published.

## Data Availability

The majority of data processing was done using Microsoft Excel 2010® version 14.0.4734.100. Python v3.8 was used for the calculations of the percentage size fractions of Chl-a, seasonal averaging (*i.e*. binning values into a monthly average for each sampled depth) and scaling the figure colormaps. The specific code written for this manuscript can be found within the plotting script at the following open source GitHub repository: https://github.com/bjmcnabb/Saanich_Inlet.
